# Exogenous tumour necrosis factor α induces suppression of autoimmune arthritis

**DOI:** 10.1186/ar2393

**Published:** 2008-04-01

**Authors:** Eugene Y Kim, Howard H Chi, Rajesh Rajaiah, Kamal D Moudgil

**Affiliations:** 1 Department of Microbiology and Immunology, University of Maryland School of Medicine, Baltimore, MD, USA.; 2 Division of Rheumatology, Department of Medicine, University of Maryland School of Medicine, Baltimore, MD, USA.

## Abstract

**Introduction:**

Our previous studies showed that arthritic Lewis (LEW) rats produced the highest levels of tumour necrosis factor (TNF)α in the recovery phase of adjuvant arthritis (AA), suggesting a correlation between high TNFα levels and reduced severity of arthritis. To further explore this correlation, we compared the TNFα secretion profile of the AA-resistant Wistar Kyoto (WKY) rats with that of LEW rats, determined the effect of exogenous TNFα on the course of AA in LEW rats, and examined various mechanisms involved in TNFα-induced disease modulation.

**Methods:**

A cohort each of LEW and WKY rats was immunised subcutaneously with heat-killed *Mycobacterium tuberculosis *H37Ra (Mtb). At different time points thereafter, subgroups of rats were killed and their draining lymph node cells were tested for cytokine production. Another group of LEW rats was injected with TNFα intraperitoneally daily for a total of 10 injections, 3 before and 6 after Mtb challenge, and then observed for signs of AA. In parallel, TNFα-treated rats were examined for changes in other cytokines, in CD4+CD25+ T cell frequency, and in indoleamine 2,3-dioxygenase (IDO) mRNA expression levels.

**Results:**

LEW rats displayed a TNFα secretion profile that was opposite to that of the WKY rats. Furthermore, TNFα treatment significantly downmodulated the severity of AA in LEW rats, and decreased the interferon (IFN)-γ secretion in response to the pathogenic determinant of the disease-related antigen. No significant alterations were observed in other parameters tested.

**Conclusion:**

The role of endogenous TNFα in the induction and propagation of arthritis is well established. However, exogenous TNFα can downmodulate the course of AA, displaying an immunoregulatory functional attribute of this cytokine.

## Introduction

Rheumatoid arthritis (RA) is a chronic autoimmune disease characterised by symmetrical joint involvement, synovial hyperplasia, neovascularisation, infiltration of the cartilage and subchondral bone by the pannus tissue leading to erosions and deformities [1-4]. Macrophages and T cells play a critical role in initiating and propagating the disease process. The cytokines tumour necrosis factor α (TNFα) and interleukin-1 (IL-1) mediate many of the inflammatory and tissue-damaging activities within the joint [1-3,5]. The *in vivo *neutralisation of these cytokines using the appropriate antibodies or decoy receptors leads to significant amelioration of signs and symptoms of joint inflammation [2,6,7]. Specifically, therapeutic strategies based on anti-TNFα antibodies or soluble TNFα receptor (sTNFR) are currently being used in clinics for the treatment of RA patients [7].

In the course of our preliminary studies in the rat adjuvant-induced arthritis (AA) model of human RA [8-13], we observed that the levels of TNFα produced by the arthritogenic epitope of mycobacterial heat-shock protein 65 (Bhsp65) [10-12,14] were highest in the recovery phase of the disease compared to that at the onset or the peak phase of AA. This unexpected correlation has formed the basis of subsequent experiments described in the present work.

Our results show that the AA-susceptible Lewis (LEW) rats given an arthritogenic stimulus (immunisation subcutaneously with heat-killed *Mycobacterium tuberculosis *H37Ra, Mtb) showed the highest levels of TNFα in the recovery phase of AA, displaying a TNFα profile opposite to that of the AA-resistant Wistar Kyoto (WKY) rats. Intriguingly, the pre-treatment of LEW rats with TNFα injected intraperitoneally induced protection against AA. This protection was attributable in part to a significant reduction of interferon (IFN)-γ production by the T cells against the arthritogenic epitope 177 to 191 of Bhsp65 (B177). However, TNFα treatment did not have a significant effect on IL-17 production [15,16], on the frequency of CD4+CD25+Foxp3+ T cells (Treg) [4,17,18], or on the level of expression of mRNA for indoleamine 2, 3-dioxygenase (IDO), the enzyme involved in tryptophan-mediated tolerogenic pathway [19,20]. Our results highlight a paradoxical arthritis-regulatory function of exogenous TNFα.

## Materials and methods

### Animals

Lewis (LEW/Hsd) (RT.1^l^) and Wistar-Kyoto (WKY/NHsd) (RT.1^l^) rats were purchased from Harlan Sprague-Dawley (HSD) (Indianapolis, IN, USA and Madison, WI, USA, respectively). Male, 4 to 6-week-old rats were used in this study. These rats were housed in the vivarium of the University of Maryland School of Medicine, Baltimore, MD, USA (UMB) and were treated as per the guidelines of the institutional animal care and use committee (IACUC) of UMB (protocol no. 0206011).

### Antigens, mitogen and cytokine

Mycobacterial hsp65 (Bhsp65) peptides 177 to 191 (B177) and 333 to 347 (B333), and HEL peptide 65 to 78 (HEL65) were obtained from Macromolecular Resources and Global Peptide Services (both at Fort Collins, CO, USA) [21,22]. The recombinant Bhsp65 was expressed and purified, as well as rendered free of endotoxin as described elsewhere [21,22]. Hen egg white lysozyme (HEL) and Concanavalin A (Con A) were purchased from Sigma-Aldrich Co. (St Louis, MO, USA), whereas purified protein derivative (PPD) was obtained from Mycos Research (Fort Collins, CO, USA). Recombinant rat TNFα was purchased from R&D Systems (Minneapolis, MN, USA), and its endotoxin content was below 1 endotoxin unit (EU)/μg. Units of TNFα were determined as ED_50 _(1 U) = 15 pg.

### Induction and evaluation of AA

LEW rats were immunised subcutaneously at the base of the tail with heat-killed *M. tuberculosis *H37Ra (Mtb) (Difco, Detroit, MI, USA) (1 mg/rat) suspended in oil (Sigma-Aldrich). Beginning on day 7 after Mtb challenge, these rats were observed and graded regularly for the severity of arthritis on the basis of erythaema and swelling of the paws on a scale of 0 to 4 as described elsewhere [12,22]. The highest arthritic score was 4 for each paw, with a maximum score of 16 per rat. Different phases of AA were labelled as follows: incubation (Inc), onset (Ons), peak (Pk), and recovery (Rec) phase.

### Lymph node cell (LNC) proliferation assay

Arthritic LEW rats were killed at different phases of AA (Inc, Ons, Pk, and Rec) and their draining lymph nodes (para-aortic, inguinal, and popliteal) were harvested post-Mtb challenge. For comparison, LNC of WKY rats immunised with Mtb were harvested at the time points corresponding to different phases of AA in LEW rats. Thereafter, a single-cell suspension of LNC was prepared, and the cells were washed three times with Hank's balanced salt solution (Invitrogen, Frederick, MD, USA) [12,22]. These LNC were cultured (2.5 × 10^5 ^cells/well) for 4 days with or without antigen at 37°C in an atmosphere of 95% air and 5% CO_2 _in a flat-bottomed 96-well plate in HL-1 serum-free medium (Ventrex Laboratories, Portland, ME, USA), which was supplemented with 2 mM L-glutamine, 100 U/ml penicillin G sodium, and 100 μg/ml streptomycin sulfate. HEL, HEL65, or B333 served as negative control antigens, whereas Con A or PPD was used as a positive control. The antigens were used at a pre-titred final concentration of 25 ug/ml that was determined to be optimal for comparison through pilot experiments. After 4 days of culture, the cells were pulsed with 1 μCi/well of [^3^H]-thymidine (International Chemical and Nuclear, Irvine, CA, USA) and then harvested after 16 to 18 h. The results were expressed either as counts per minute (cpm) or as a stimulation index (SI = cpm of cells cultured with antigen/cpm of cells in medium alone).

### Collection of supernatant from LNC culture and testing for cytokines by enzyme-linked immunosorbent assay (ELISA)

The LNC harvested from Mtb-immunised LEW and WKY rats were cultured in a 96-well plate as described above. These LNC were then re-stimulated *in vitro *for 48 to 72 h with the appropriate antigen, and the culture supernates were collected thereafter [22]. These supernates were then tested by ELISA using commercially available kits for the detection of TNFα, IFN-γ and IL-10 (all from Biosource, Camarillo, CA, USA), with lower detection limits (pg/ml) of 4, 13, and 10, respectively. The results were expressed as pg/ml. For comparison of different groups, the background cytokine level was deducted from the antigen-specific cytokine secretion (pg/ml of cytokine from cells cultured with antigen – pg/ml of cytokine from cells in medium alone; also referred to as Δ pg/ml) [22,23].

### Modulation of AA by *in vivo *TNFα treatment of LEW rats

TNFα was injected intraperitoneally daily into naive LEW rats at 1 × 10^5 ^U/ml per injection beginning 3 days before immunisation subcutaneously with Mtb on the fourth day. TNFα treatment was continued through 6 days post Mtb injection for a total of 10 injections. Control rats received equal number of injections of phosphate-buffered saline (PBS) following the same protocol as that used for TNFα injections, including Mtb injection after 3 days of starting PBS injection. Thereafter, all rats were observed regularly for signs of arthritis, and the severity of the disease was scored as described above.

### Collection of sera and their testing for sTNFR-I and anti-TNFα antibody by ELISA

Blood from LEW rats treated with TNFα *in vivo *as described above along with that from control rats was collected either from tail vein or via cardiac puncture. The serum was separated from the clotted blood and tested in ELISA for the presence of sTNFR-I or anti-TNFα antibody. ELISA for sTNFR-I (R&D Systems, Minneapolis, MN, USA) was performed following the manufacturer's instructions, and the results were expressed as pg/ml. ELISA for anti-TNFα antibody was set up and optimised in-house. The ELISA plate (Greiner, Monroe, NC, USA) was coated with 100 μl (0.1 μg/well) of TNFα (Biosource, Camarillo, CA, USA) overnight at 4°C. After washing with PBS containing 0.05% Tween-20 (PBST), the wells were blocked with 200 μl/well of 10% bovine serum albumin (BSA) in PBST. Thereafter, the plate was washed, and 100 μl of diluted rat sera (1:50, 1:100, 1:200, and 1:400) were added per well and incubated at room temperature for 1 h. After washings, 100 μl of horseradish peroxidase (HRP)-conjugated polyclonal anti-rat antibody (BD PharMingen, San Diego, CA, USA) (1:2,500) was added per well. After 1 h at room temperature, the plate was washed, and the colour was developed by adding 30 μl/well of ABTS substrate (Bio-Rad, Hercules, CA, USA) and incubating for 15 min. The colour reaction was then stopped with 50 μl/well of 0.5 M H_2_SO_4_. The OD_450 _was measured using a Vmax microplate reader (Molecular Devices, Sunnyvale, CA, USA).

### Flow cytometric analysis of CD4^+^Foxp3^+ ^Treg and peritoneal lavage cells

#### CD4+Foxp3+ T cells

TNFα-treated and Mtb-immunised LEW rats (test group) were bled before and after the set of 10 injections of TNFα, and the blood samples were collected under heparin. Thereafter, the red blood cells (RBC) were lysed with ACK lysis buffer (Sigma-Aldrich), and the remaining cells were surface-stained first with anti-rat CD4-FITC (BD Biosciences, San Jose, CA, USA), followed by permeabilisation and staining with anti-mouse/rat Foxp3-PE (eBioscience, San Diego, CA, USA) [17,18]. These stained cells were then analysed by fluorescence-activated cell sorting (FACS) using the FACS Caliber and CellQuest software (both from BD Biosciences). A similar procedure was followed when using LNC and spleen cells.

#### Peritoneal lavage cells

LEW rats were injected intraperitoneally daily for 4 days either with TNFα or with PBS. The peritoneal cavity of these rats was then flushed with PBS 3 h after the last injection, and 10 ml of lavage fluid was collected. The lavage fluid was centrifuged to collect the cells therein. These cells were then stained with labelled antibodies against CD3 or CD11b/c followed by analysis by flow cytometry.

### Determination of IL-17, IDO, and tryptophanyl-tRNA-synthetase (TTS) mRNA levels in antigen-sensitised cells by qRT-PCR

The draining LNC were harvested from TNFα- or PBS-treated and Mtb immunised rats, and cultured for 48 h in the presence or absence of the appropriate antigen. Total RNA was prepared from 1 × 10^6 ^cells and reverse-transcribed using the iScript cDNA synthesis kit (Bio-Rad Laboratories). The cDNA thus obtained was amplified using an ABI Prism 7900HT cycler (Applied Biosystems, Foster City, CA, USA) [24]. The primers used in the assay for the detection of mRNAs for IL-17, IDO, TTS, and hypoxanthine-guanine phosphoribosyl transferase (HPRT) were designed using the Primer Express 2.0 program (Applied Biosystems) and were synthesised at the UMB Biopolymer Core Facility. The mRNA levels of each entity tested were normalised to the *HPRT *gene, and the relative gene expression levels were determined [24]. The results were expressed as 'fold increase' over mRNA levels of cells cultured in medium alone. We also confirmed the IDO mRNA expression results in splenic adherent cells (macrophages and dendritic cells).

### Statistical analysis

The Student t test assuming equal or unequal variance (determined by the F test) was used as appropriate for the data to test the statistical significance of the differences observed among various test and control groups. A non-parametric Wilcoxon rank sum test was employed to compare the arthritic scores of any two groups of rats over the entire disease course. The results were considered significant at p < 0.05.

## Results

### Arthritic LEW rats show highest levels of TNFα at the recovery phase of AA, whereas AA-resistant WKY rats exhibit an opposite profile

**Figure 1:**
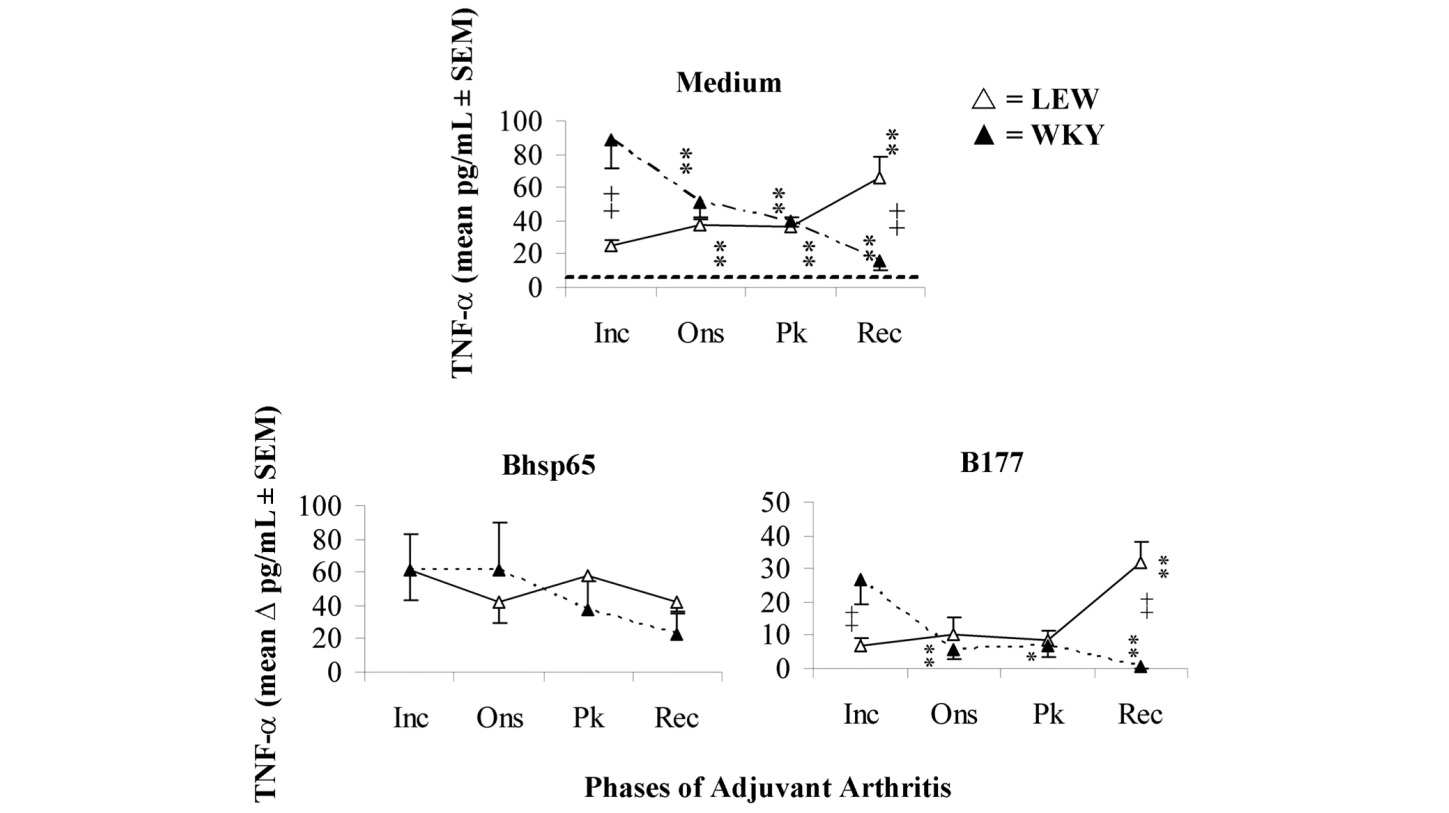
*Mycobacterium tuberculosis *H37Ra (Mtb)-immunised Lewis (LEW) rats showed the highest level of tumour necrosis factor (TNF)α secretion during the Rec phase of adjuvant arthritis (AA), but Wistar-Kyoto (WKY) rats displayed an opposite profile. LEW (△) (n = 4 each) and WKY (▲) (n = 3 each) rats were killed at different time points after Mtb injection and their draining lymph node cells (LNC) were harvested. These LNC were cultured for 48 h in a 96-well plate with or without the addition of any exogenous antigen. The supernates were then collected and analysed for TNFα by enzyme-linked immunosorbent assay (ELISA). The LNC/culture supernates of individual rats were tested separately and then the results of each of the two subgroups (LEW/WKY) were presented as pg/ml (mean ± SEM). For comparison, medium background was subtracted from antigen-induced cytokine (Δ pg/ml). *p < 0.05 and **p ≤ 0.025, when levels of a particular cytokine at other phases of AA were compared with that at Inc phase for the same rat strain (LEW/WKY); +, p ≤ 0.05, and ++, p ≤ 0.025, when cytokine levels were compared between LEW and WKY rats at the corresponding phase of AA. Inc = incubation phase; Ons = onset phase; Pk = peak phase; and Rec = recovery phase. Testing of additional animals following the above protocol yielded similar results.

The results of *ex vivo *TNFα secretion (cytokine secretion without any exogenously added antigen; Figure 1) showed that there was a gradual increase in levels along with the progression (time post-Mtb injection) of AA in LEW rats with the highest level observed during Rec phase, while an opposite pattern was observed in WKY rats. However, following re-stimulation with Bhsp65, TNFα secretion was at a high level in both LEW and WKY rats without significant changes during the course of AA (Figure 1). Importantly, the level of TNFα secreted in response to the pathogenic epitope B177 of Bhsp65 was significantly increased at the Rec phase of AA in the LEW rats, but at Inc phase in WKY rats. Overall, the highest level of TNFα secretion was observed during Rec phase in LEW rats, but at Inc phase in WKY rats.

### The severity of AA is downmodulated following *in vivo *TNFα treatment of LEW rats

**Figure 2:**
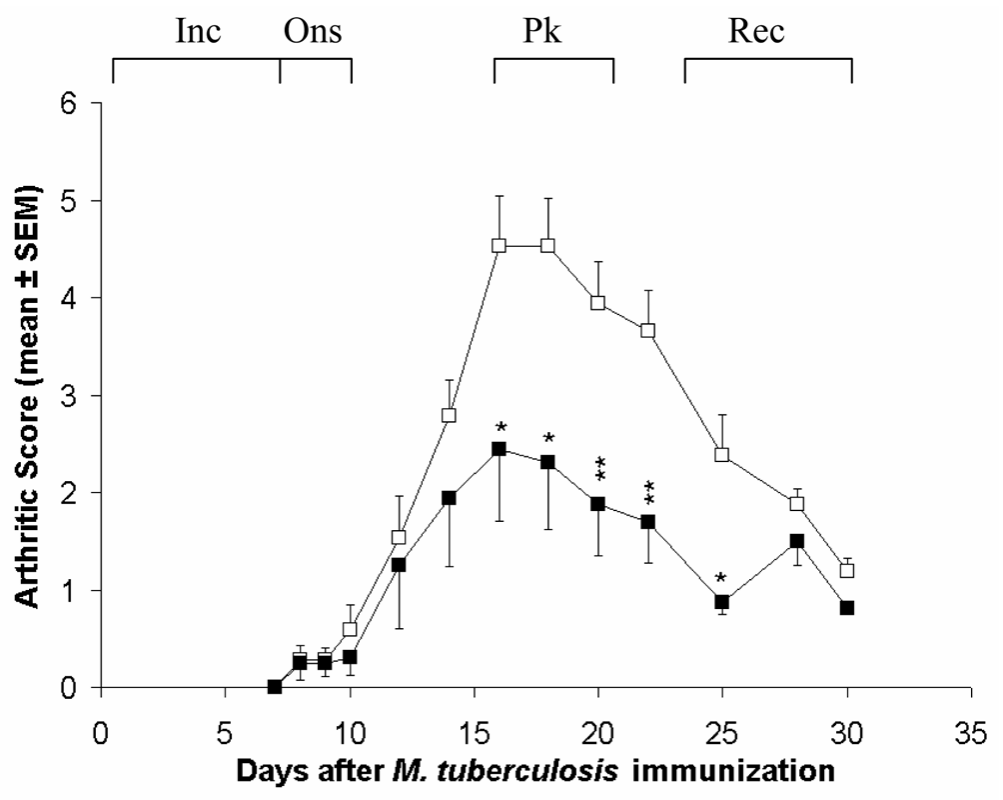
Downmodulation of adjuvant arthritis (AA) by *in vivo *tumour necrosis factor (TNF)α treatment of Lewis (LEW) rats. LEW rats were injected intraperitoneally daily either with 1 ml of 10^5 ^U/ml TNFα (n = 4; experimental group; ■) or with 1 ml PBS (n = 8; controls; □) for 3 days before the day of *Mycobacterium tuberculosis *H37Ra (Mtb) injection, and then continued daily for 7 days, including the day of Mtb injection, to a total of 10 injections. Thereafter, all rats were observed for signs of AA, and the severity of arthritis was graded as described in Materials and methods. The difference in the severity of arthritis during the course of AA in the two groups of rats was statistically significant from day 16 through day 25 (*p < 0.05; **p < 0.025). The difference between the two rat groups was also significant (p < 0.05) when analysed by Wilcoxon rank sum test. Similar results were obtained in repeat experiments. Also shown in the figure is a representative designation of different phases of the disease in the course of AA in the form of days post-Mtb immunisation as follows: Inc = incubation, days 1 to 7; Ons = onset, days 8 to 10; Pk = peak, days 15 to 18; and Rec = recovery, days 23 to 30.

The above results showed that high TNFα levels correlate with recovery from acute AA in LEW rats, and with resistance against AA in WKY rats. To further examine this correlation, we tested the effect of TNFα treatment on AA in the LEW rats. Naïve LEW rats were given a total of 10 injections intraperitoneally of TNFα (10^5 ^U/day) in PBS with three doses given before Mtb-injection and then continued on the day (fourth day) of Mtb injection and for 6 more days thereafter. After Mtb injection, rats were observed regularly for signs of arthritis. The control rats received 10 injections of PBS and were injected with Mtb at the same time as the experimental rats. The results (Figure 2) revealed that the TNFα-treated rats had a significantly reduced severity of AA compared to that of the control rats. This suppression of AA in the experimental group of rats was evident before the peak of AA, and it continued for an average of 7 days. Thus, treatment with TNFα, a pro-inflammatory cytokine, significantly attenuated the severity of AA in the LEW rats.

### *In vivo *TNFα treatment of Mtb-immunised LEW rats decreases IFN-γ secretion in response to the pathogenic determinant B177 of Bhsp65

**Figure 3:**
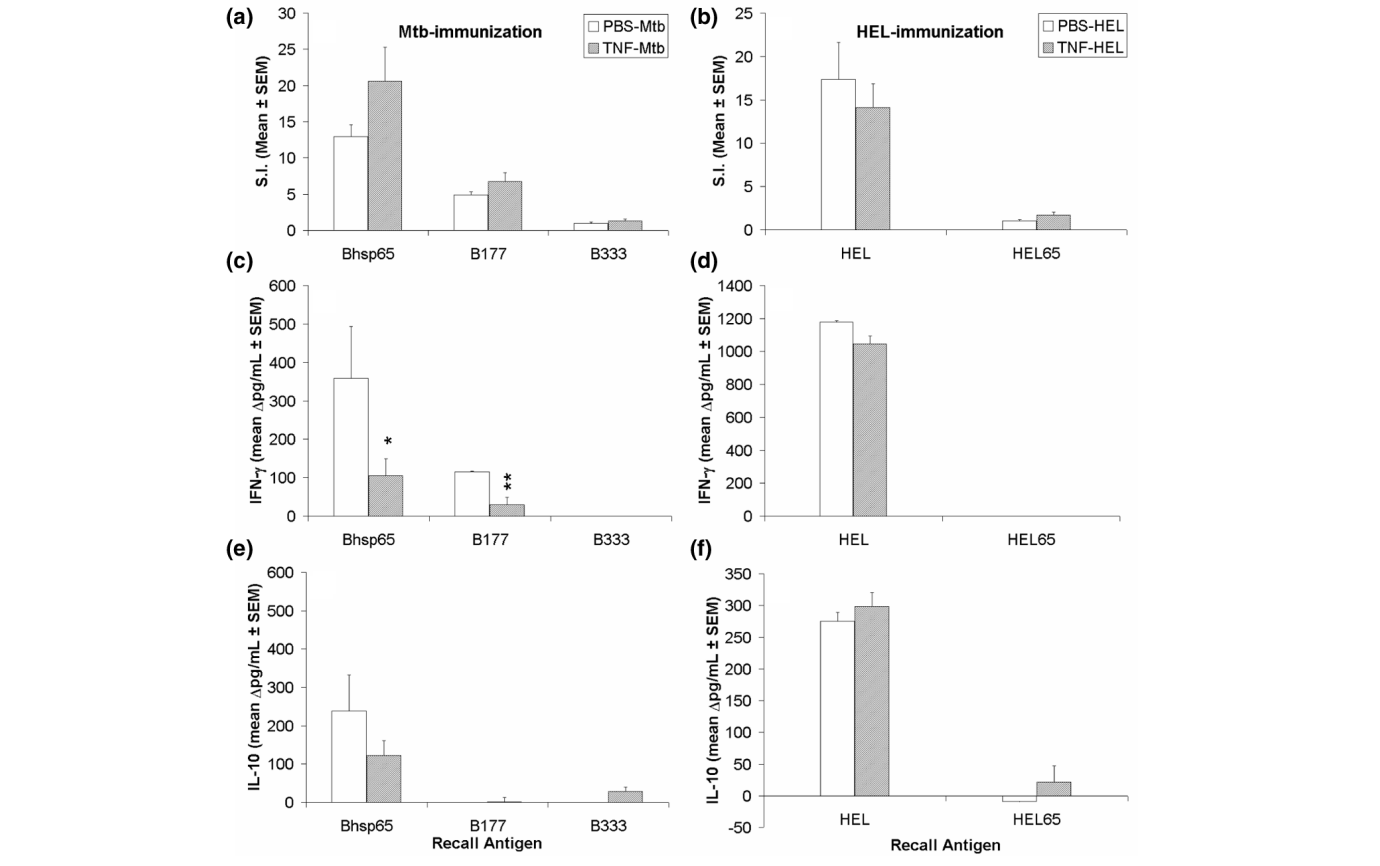
*In vivo *tumour necrosis factor (TNF)α treatment resulted in decreased interferon (IFN)-γ secretion by B177-restimulated LNC without affecting their proliferative response. LEW rats were treated with PBS (□) or TNFα () as described in the legend to Figure 2, with the exception that one subgroup of rats was immunised with *Mycobacterium tuberculosis *H37Ra (Mtb) **(a, c, e)**, whereas the other was injected with HEL/IFA **(b, d, f)**. At day 9 after injection with Mtb or HEL, the draining LNC of these rats were harvested and tested in a proliferation assay (**(a, b)**; n = 8 each). Peptide 333 to 347 of Bhsp65 (B333), peptide 65 to 78 of HEL (HEL65), and native HEL were used as control peptide/protein antigens. The results are presented as mean stimulation index (SI) ± SEM. In addition, the supernates collected after 72 h of culture of LNC of Mtb- or HEL-immunised rats were tested by ELISA for IFN-γ **(c, d) **and interleukin (IL)-10 **(e, f) **(n = 5 each). The results of cytokine analysis are shown as Δ pg/ml (mean ± SEM). *, p < 0.05 and **, p ≤ 0.025, when compared with the respective PBS control.

As TNFα treatment decreased the severity of AA, we tested whether the suppression of AA involved any major changes in the immune responsiveness to antigenic challenge. LEW rats were treated with TNFα using the protocol described above, including immunisation with Mtb or a control antigen (HEL/IFA). After 9 days of antigenic challenge, the draining LNC of these rats were harvested and tested for proliferative and cytokine response using Bhsp65, HEL, using their peptides as recall antigens. We obtained comparable (p > 0.05) numbers of LNC from TNFα-treated and PBS-treated rats in both Mtb-immunised and HEL-immunised groups (data not shown), suggesting that, at the dose used, the injected TNFα did not lead to a significant change in the number of cells (for example, via apoptosis) in the draining lymph nodes. In the cohort of Mtb-immunised rats, the LNC recall response to Bhsp65 and B177 in TNFα-treated rats was comparable to that of PBS-treated control rats (Figure 3a). Similar results were obtained in the two groups of rats that were immunised with HEL (Figure 3b) instead of Mtb. Furthermore, the results of cytokine testing showed that IFN-γ secretion by LNC of TNF-treated, Mtb-immunised LEW rats after B177 recall decreased significantly compared to that of the PBS-treated control rats (Figure 3c). This decrease in IFN-γ secretion in Mtb-immunised LEW rats was specific to B177 as IFN-γ response to the control antigen (HEL) in PBS-treated, HEL-immunised rats was comparable to that of TNFα-treated, HEL-immunised rats (Figure 3d). As there was no difference in the level of IL-10 secretion between the two Mtb-immunised groups (Figure 3e,f), the decrease in IFN-γ in response to B177 in TNFα-treated, Mtb-immunised LEW rats steered the overall cytokine response towards a T_h_2 type. Thus, TNFα treatment neither resulted in a general non-specific enhancement of antigen-specific proliferative T cell response, nor induced a generalised immunosuppression. Instead, the AA-protective effect of TNFα involved a decrease of IFN-γ in response to the pathogenic epitope (B177) of Bhsp65.

In another set of experiments, we examined whether TNFα treatment had any effect on IL-17 production by Bhsp65- or B177-reactive T cells. We tested IL-17 by qRT-PCR because of rather limited reagents for the newer rat cytokines, including IL-17. The level of IL-17 in TNFα-treated, Mtb-immunised rats was comparable (p > 0.05) to that of PBS-treated, Mtb-immunised rats (data not shown).

### TNFα-treatment does not lead to any changes in serum levels of sTNFR-I and anti-TNFα antibody

**Figure 4:**
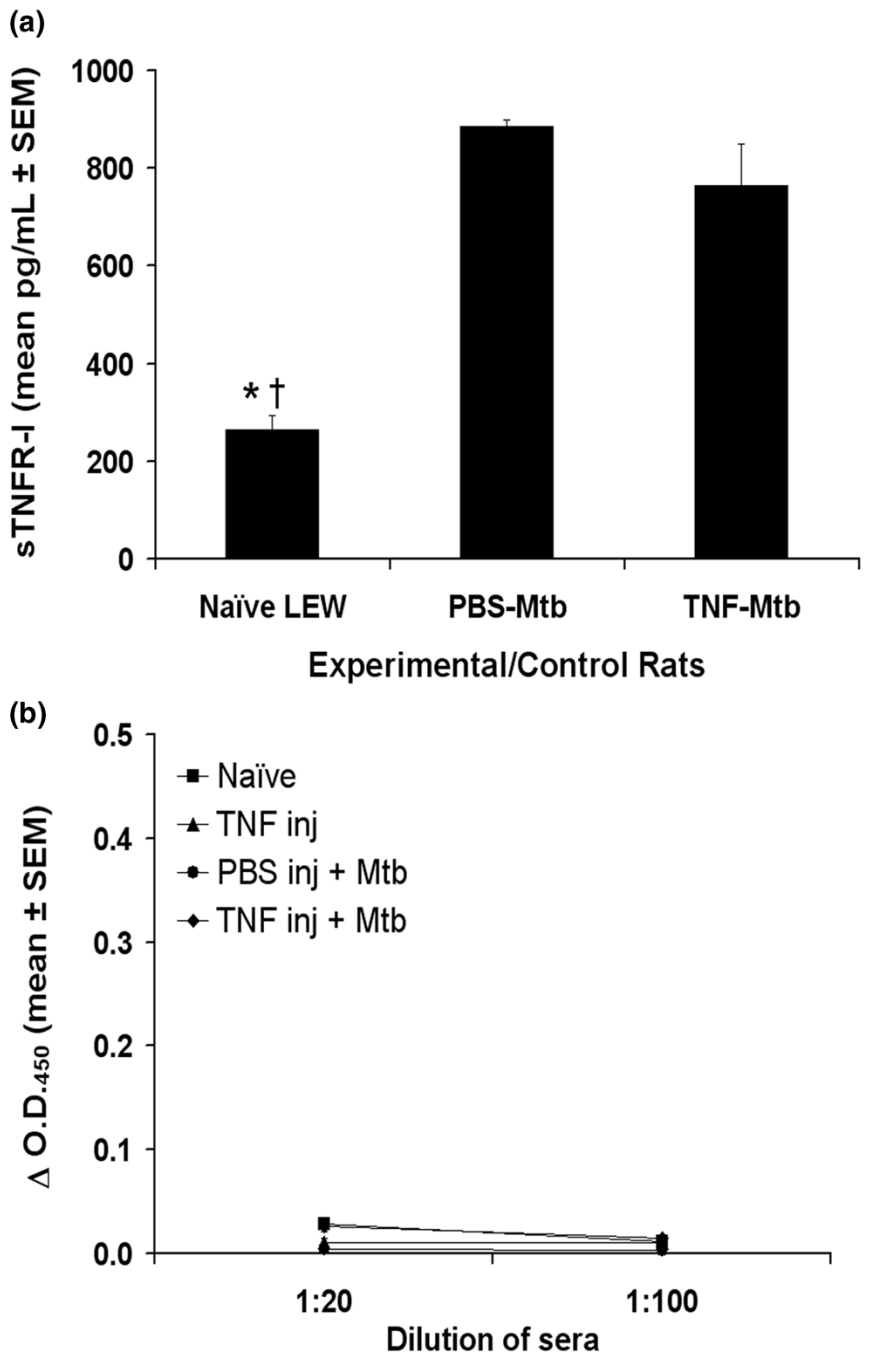
Tumour necrosis factor (TNF)α treatment of Lewis (LEW) rats neither increased the release of soluble TNF receptor I (sTNFR-I) nor induced the generation of anti-TNFα antibody. LEW rats were injectedintraperitoneally daily with 1 ml of either 10^5 ^U/ml TNFα or phosphate-buffered saline (PBS) for 3 days before *Mycobacterium tuberculosis *H37Ra (Mtb) immunisation, and then continued daily for a total of 10 injections. At day 9 after Mtb immunisation, blood samples were collected from these rats. The sera were then tested for sTNFR-I (A; n = 3+) and anti-TNFα antibody (B; n = 3+) by enzyme-linked immunosorbent assay (ELISA). Appropriate positive controls gave optimal results. The results of sTNFR-I are presented as mean pg/ml ± SEM, and the results of anti-TNFα antibody are presented as OD_450 _(mean ± SEM). *p < 0.05 and †p ≤ 0.05, when naïve sera was compared with the PBS injected-Mtb sera and TNF injected-Mtb sera, respectively.

We examined two other parameters that might contribute to TNFα-mediated protection against AA. First, the excessive shedding of sTNFR-I [25,26], and second, the generation of anti-TNFα antibody that might neutralise TNFα *in vivo *[27]. The levels of sTNFR-I (Figure 4A) as well as anti-TNFα antibodies (Figure 4B) in sera of TNFα-treated, Mtb-immunised LEW rats were comparable to that in sera of PBS-treated, Mtb-immunised LEW rats.

### TNFα injections intraperitoneally do not induce any preferential cell migration into the peritoneum

**Figure 5:**
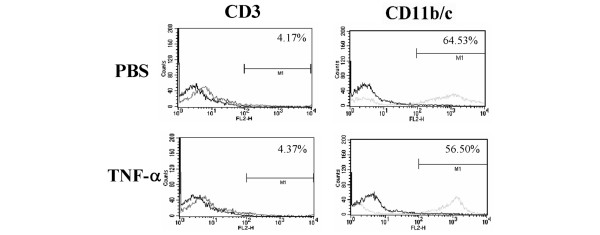
The composition of peritoneal lavage cells of tumour necrosis factor (TNF)α-treated Lewis (LEW) rats was comparable to that of phosphate-buffered saline (PBS)-treated rats. LEW rats (n = 4) were injected intraperitoneally daily with 1 ml of PBS (top panel) or TNFα (10^5 ^U) (bottom panel) for 4 days. After 3 h post the fourth injection, the peritoneal cavity was flushed with PBS and 10 ml of the peritoneal lavage fluid was collected. The cells harvested from the lavage fluid were stained with appropriately labelled anti-CD3 or anti-CD11b/c antibody and analysed by fluorescence-activated cell sorting (FACS). The results of one of the two independent experiments are shown in the figure. Both experiments yielded similar results.

We also tested whether intraperitoneal injection of TNFα might deviate the migration of T cells away from the joints into the peritoneal cavity. Our results showed no difference in the number/proportion of T cells (CD3) or macrophages/neutrophils (CD11b/c) infiltrating into the peritoneal cavity after PBS treatment vs TNFα treatment intraperitoneally (Figure 5). These results suggest the absence of a major shift in the migration of T cells into the peritoneal cavity following TNFα treatment.

### The level of expression of mRNA for IDO as well as the frequency of CD4+Foxp3+ T cells (Treg) is unaltered by TNFα treatment

To gain further insights into the mechanisms by which TNFα treatment might suppress AA, we compared the relative levels of components of the two immunosuppressive pathways, the IDO-TTS by qRT-PCR and the Treg by flow cytometry. IDO is predominantly expressed in myeloid cells, and it catabolises tryptophan [19,20,28]. By contrast, TTS binds to tryptophan and makes it available for protein synthesis [19,20,28]. The IDO-induced deprivation of tryptophan has been invoked in T cell tolerance and suppression of T cell response. Similarly, Treg can suppress the activity of pathogenic effector T cells via cell-cell contact and immunomodulatory cytokines, TGF-β and IL-10 [17,18]. Our results show that the levels of IDO mRNA in Bhsp65-restimulated LNC of TNFα-treated rats (1.51 fold compared to LNC in medium) were comparable (p > 0.05) to that of PBS-treated rats (2.26 fold). Similarly, the TTS mRNA levels in TNFα-treated versus PBS-treated rats were 1.69 fold versus 2.6 fold, respectively, and this difference was not significant (p > 0.05). The results for IDO mRNA testing using splenic adherent cells (data not shown) were similar to that obtained with LNC. In regard to Treg frequency, the levels (mean ± SEM) were slightly lower in TNFα-treated rats (8.5% ± 0.4) than that of PBS-treated rats (10.2% ± 0.3), but this difference was not statistically significant (p > 0.05).

## Discussion

We observed that TNFα secretion in response to the arthritogenic epitope of Bhsp65 (B177) during the course of AA in the LEW rat showed a paradoxically opposite profile in relation to the disease severity. Considering the critical role of TNFα in the initiation and propagation of arthritis, we had anticipated that the level of TNFα might be high in the early phases of AA (for example, Inc, Ons, and/or Pk), but relatively much lower in the later phases (for example, Rec) of the disease. However, the actual picture that was revealed was surprisingly reverse, in that the arthritic LEW rats showed highest TNFα secretion in the Rec phase of the disease compared to that at Ons or Pk. This association of high TNFα levels with the decline of inflammatory arthritis was also supported by the TNFα secretion profile of the AA-resistant WKY rats. Unexpectedly, the WKY rats secreted high levels of TNFα early after Mtb challenge, and the TNFα secretion then gradually declined with time post-Mtb challenge, showing the reverse association of disease activity/severity vs TNFα levels produced in response to the pathogenic epitope of Bhsp65. However, this negative correlation suggests but does not establish a causal relationship between endogenous TNFα and protection against arthritis. In this regard, our results of suppression of AA by exogenous TNFα suggest that this cytokine also possesses an immunoregulatory component. It is conceivable that the conditions under which the same cytokine would manifest differential functional activities (pathogenic vs regulatory) might be distinct, and these conditions have yet to be fully defined. We propose that the concentration of TNFα is one of the critical factors influencing the predominantly pathogenic vs protective effect of the cytokine. Some of these factors are also revealed in studies based on anti-TNF therapy in the AA model. Soluble TNF-receptor (sTNF-RI) administered to LEW rats on days 9, 11, and 13 of AA led to inhibition of AA, and the level of suppression was dose-dependent [29]. Similarly, the treatment of rats with sTNF-RI beginning on day 4 after disease onset induced suppression of AA [30]. By contrast, in another study, sTNF-RI treatment of DA rats on days 0, 2, and 4 post-Mtb injection had no significant effect on early phase of AA [31]. However, later in the course of AA, lower dose of sTNF-RI exacerbated AA, while higher dose failed to alter the disease severity, supporting a concentration-dependent biologic effect of this pro-inflammatory cytokine [31]. In a study conducted in the CIA model, adenovirus-mediated gene delivery of TNFR-IgG fusion protein initially suppressed arthritis but subsequently exacerbated the disease [32]. Taken together, these studies highlight both the disease-aggravating and the disease-suppressing effects of TNFα.

We described above that systemic administration of TNFα into LEW rats can downmodulate the course of clinical AA. We ruled out the induction of any generalised immunosuppression due to chronic TNFα treatment by showing that TNFα-treated, HEL-immunised LEW rats raised a robust proliferative and cytokine response to the immunogen. Moreover, we demonstrated that TNFα-treated LEW rats showed a significant decrease in IFN-γ secretion in response to B177 without much change in the proliferative response to the same antigen. The ratio of IFN-γ to IL-10 showed a decrease, but this skewing of the cytokine response was mainly because of a decrease in IFN-γ levels rather than an increase in IL-10 secretion. This decrease in IFN-γ levels could occur in part via TNFα-mediated negative regulation of IL-12 production [33]. Although IFN-γ and TNFα are both pro-inflammatory cytokines, but these cytokines might be regulated by different mechanisms and also trigger differential effects [34] in a concentration-dependent mechanism. The downregulation of IFN-γ production by the T cells following chronic TNFα exposure has also been reported by other investigators [35]. However, unlike for IFN-γ, we did not observe a significant change in IL-17 response of Bhsp65- or B177-reactive T cells following TNFα treatment. As Th1 and Th17 subsets of T cells are distinct lineages in regard to their differentiation and regulation by different cytokines, a change in the production of one (IFN-γ) but not the other (IL-17) cytokine after TNFα treatment of rats is not an unexpected finding.

We also considered the earlier results of other investigators showing that TNFα treatment can induce the shedding of soluble TNF receptor I (sTNFR-I) from cell surface, which in turn can bind circulating TNFα and suppress signals for continuation of inflammation [25,26]. However, our analysis of sTNFR-I in the sera of TNF-treated, Mtb-immunised LEW rats excluded any significant change in sTNFR-I levels compared to that of PBS-treated, Mtb-immunised LEW rats. Similarly, we also ruled out the presence of circulating anti-TNFα antibodies in the serum following TNFα injection, which in turn could neutralise TNFα *in vivo*. We also excluded a major shift in the migration of subsets of mononuclear cells into the peritoneal cavity following TNFα injection intraperitoneally. Similarly, we also ruled out any TNFα-induced enhancement of the level of mRNA for IDO, the enzyme involved in IDO-tryptophan tolerance pathway and the level of CD4+CD25+ T cells (Treg). In this study, we have tested only IDO mRNA expression but not the IDO enzyme activity. Other investigators have demonstrated that the induction of IDO activity is a two-step process, with prostaglandin E2 causing an increase in IDO expression and TNFα (or toll-like receptor ligands) leading to an increase in IDO enzymatic activity [36]. Therefore, the precise contribution of IDO-tryptophan pathway to the TNFα-induced suppression of AA needs to be further explored. In regard to Treg, there are limited reports on the effects of TNFα on Treg frequency, and these revealed contrasting effects [37-39]. However, in RA patients, an increase in Treg numbers with anti-TNFα treatment has been reported [38,40], which indirectly supports our observed trend (but not significant) towards decreased Treg numbers in TNFα-treated rats. TNFα may also influence other important functions *in vivo *that have not been addressed at this time in our study; for example, (a) apoptosis within the target organ of pathogenic T cells that mediate arthritis induction [41,42]; (b) alteration of the migration of inflammatory cells into the joints by changing the expression of adhesion molecules on endothelial cells [43]; (c) triggering of the HPA axis by elevated systemic TNFα, leading to the release of corticosteroids and suppression of TNFα in the target organ (the joints) [44,45]; (d) the induction of immunoregulatory cytokine IL-10, leading to the suppression of pathogenic TNFα [46-48]; and (e) the modulation of dendritic cells *in vivo*, which then present antigen favouring downregulation of arthritis [49].

Our results highlight the immunoregulatory role of exogenous TNFα in AA. Immune regulation by TNFα has been observed in other models of autoimmune diseases as well. For example, the downregulation of type 1 diabetes (T1D) in the non-obese diabetic (NOD) mouse by CFA immunisation has been shown to involve TNFα production and granzyme B/perforin-secreting Treg [42,50]. In another study, TNFα expression within the pancreas prevented diabetes in NOD mice [51], while systemic treatment of TNFα in adult NOD mice decreased insulitis as well as the incidence of diabetes [52]. However, the modulation of diabetes by TNFα is influenced significantly by the timing of administration or of the *in vivo *expression of TNFα during the disease pathogenesis [53,54]. Similarly, using the myelin oligodendrocyte glycoprotein (MOG)-induced experimental autoimmune encephalomyelitis (EAE) model, it has been shown that TNFα KO mice developed more severe EAE, while TNFα treatment ameliorated the disease [55]. Furthermore, studies in myocarditis have highlighted the pathogenic as well as the protective roles of pro-inflammatory cytokines [56,57]. In RA, there are several convincing pieces of evidence to support the critical role of TNFα in mediating the autoimmune inflammation [1,3,5], and accordingly, TNFα antagonists are a significant addition to the therapeutic arsenal against RA [6,7]. However, our study has addressed the understudied and under-appreciated protective or immunoregulatory role of exogenous TNFα against autoimmune arthritis. These results have implications on our understanding of the complex processes involved in the pathogenesis of autoimmune arthritis as well as on the full range of effects on immune responsiveness of individuals receiving anti-TNFα agents for arthritis and other clinical conditions.

## Conclusion

Pre-treatment of LEW rats with TNFα downmodulated the severity of AA, and this TNFα induced protection against arthritis involves suppression of IFN-γ production by the T cells against the arthritogenic epitope of Bhsp65.

## Abbreviations

AA = adjuvant arthritis; Bhsp65 = mycobacterial heat shock protein 65; B177 = Bhsp65 peptide 177 to 191; B333 = Bhsp65 peptide 333 to 347; HEL = hen egg white lysozyme; HEL65 = HEL peptide 65 to 78; Inc = incubation; LEW = Lewis; LNC = lymph node cells; Mtb = *Mycobacterium tuberculosis *H37Ra; Ons = onset; Pk = peak; Rec = recovery; SI = stimulation index; sTNFR-I, soluble TNF receptor I; WKY = Wistar-Kyoto.

## Competing interests

The authors declare that they have no competing interests.

## Authors' contributions

EYK conducted most of the experimental work, designed experiments, recorded and analysed the raw data, participated in the interpretation of results as well as writing of the manuscript. HHC contributed to the manuscript by designing and conducting some of the experiments, and by recording, analysing, and interpreting the results of those experiments. RR designed and conducted some of the experiments, analysed and interpreted their results, and participated in the writing of the manuscript. KDM contributed by designing the experiments, by analysing and interpreting the results, by writing of the manuscript, and by arranging the grant support for this study.
